# Physical, Mechanical, Barrier, and Optical Properties of Sodium Alginate/Gum Arabic/Gluten Edible Films Plasticized with Glycerol and Sorbitol

**DOI:** 10.3390/foods14071219

**Published:** 2025-03-31

**Authors:** Adiansyah Syarifuddin, Nur Haliza, Nur Izzah, Mulyati Muhammad Tahir, Andi Dirpan

**Affiliations:** 1 Department of Agricultural Technology, Hasanuddin University, Makassar 90245, Indonesia; halizanr161@gmail.com (N.H.); nur.izzah852@gmail.com (N.I.); p.mulyati@yahoo.com (M.M.T.); dirpan@unhas.ac.id (A.D.); 2 Center of Excellence in Science and Technology on Food Product Diversification, Makassar 90245, Indonesia

**Keywords:** glycerol, sorbitol, sodium alginate, gum arabic, gluten, edible film

## Abstract

Polysaccharides and proteins are the primary components of edible films used for food packaging. Adding plasticizers such as glycerol or sorbitol during manufacturing can help enhance the properties of films derived from biopolymer combinations. In this study, we aimed to produce sodium alginate/gum arabic/gluten edible films and evaluate the effects of various concentrations of glycerol and sorbitol used as plasticizers on the films’ physical, mechanical, barrier, and optical properties. Using solvent casting, an edible film based on sodium alginate/gum arabic/gluten was plasticized with either glycerol or sorbitol at concentrations of 2, 4, and 6% (*w*/*v*). The properties of the edible films were then characterized. Decreases in solubility, tensile strength, and water vapor transmission rate were observed when higher glycerol and sorbitol concentrations were added. The films plasticized with 6% glycerol and 6% sorbitol had the lowest solubility, tensile strength, and water vapor transmission rates. In addition, the films plasticized with glycerol, regardless of concentration, had lower transparency values than those plasticized with sorbitol. The addition of glycerol and sorbitol had insignificant effects on the thickness properties and L values of the films. The absorption peaks of the Fourier-transform infrared spectra patterns of the films plasticized with sorbitol and glycerol were similar, confirming there was an interaction between the plasticizers and polymers. Together, the results demonstrate that sorbitol and glycerol are compatible with sodium alginate/gum arabic/gluten film-forming solutions, indicating that the films obtained could be employed for food packaging.

## 1. Introduction

Interest in developing edible, biodegradable, and eco-friendly materials for primary packaging has increased since petroleum-based packaging poses environmental and safety risks. Petroleum-derived packaging, like plastic, can take over 100 years to break down. During this process, it releases various harmful emissions, including carbon monoxide, hydrochloric acid, chlorine, dioxin, amines, furans, nitrides, benzene, 1-styrene, 3-butadiene, and acetaldehyde. These emissions have a detrimental impact on the environment [1,2]. Edible films constructed using biopolymers and plasticizers have recently gained a great deal of attention as a possible replacement for petroleum-based packaging. Sodium alginate and gum arabic are biopolymers that can be used to make edible films because of their high processability and abundance. Previous research has demonstrated that sodium alginate is suitable for edible film fabrication due to its mucoadhesive properties, biodegradability, biocompatibility, and cross-linking abilities. Additionally, sodium alginate can interact with other polymers or food additives, and it exhibits antibacterial and antioxidant effects [3]. Sodium-alginate-based edible films prepared with chestnut extract and mixtures of bio-based plasticizers have been shown to have better mechanical and antimicrobial properties than films created using chestnut extract and commercial plasticizers [4]. Additionally, they can maintain fresh pork’s appearance, sensory attributes, and physiological quality after 14 days of cold storage at 4 °C [5]. However, using sodium alginate as the only biopolymer in edible films presents certain drawbacks, including low elongation-at-break (EAB) values, susceptibility to breakage, and poor water resistance [6]. Various biopolymers, such as pectin, xanthan gum, gluten, and gum arabic, have been mixed into sodium alginate matrices to increase the physical, mechanical, barrier, and optical properties of sodium-alginate-based films. For example, a blend of sodium alginate and gum arabic employed to fabricate edible films through the casting method exhibited effective mixing; the gum arabic formed an amorphous structure, and the sodium alginate had a semi-crystalline nature. In addition, SEM micrographs of the blends showed complete miscibility of the two polymers [7]. Some studies have shown that edible films produced by combining sodium alginate/gum arabic/glycerol loaded with natamycin could modify the mechanical and barrier properties and thermal stability of composite films as well as delay sweet potatoes’ physiological and quality changes during traditional storage and prolong their shelf life [8]. In addition, mixing sodium alginate with pectin and xanthan gum yielded films that showed high compatibility, vapor resistance, and high efficiency, properties allowing them to be used to prolong the shelf-life of fresh-cut potatoes [9]. Moreover, sodium-alginate-based films combined with leaf extracts of *Vitis vinifera* could enhance the films’ physical, antibacterial, and antioxidant properties when an ultrasound-assisted fabrication method was used [10].

Gum arabic, a biodegradable and biocompatible polysaccharide obtained from the stems and branches of the acacia tree [11], has shown good water barrier properties and antioxidant effects when combined with chitosan in a ratio of 1:2 [12]. Galactose, rhamnose, arabinose, and glucuronic acid are considered the crucial components of this substance because of their ability to generate low-viscosity products and emulsify film-forming solutions [13]. However, films containing only gum arabic suffer from disadvantages such as poor mechanical strength and low water resistance. Therefore, researchers have combined gum arabic with other components to improve films’ physical, mechanical, and barrier properties [14]. Blending white almond isolate protein and gum arabic to create edible films increased the analyzed films’ tensile strength and elongation but decreased their water vapor permeability [15]. Moreover, using lower concentrations of gum arabic with aqueous extracts of *Syzygium aqueum*, *Diploglottis bracteata*, and *Tasmannia lanceolata* inhibited microbial growth while maintaining the sensory properties of fresh-cut red capsicum [16].

The strategy consisting of combining two polysaccharides with protein is being extensively investigated for constructing bio-based mixed films with tailored properties instead of single-component films. Combining gum arabic and sodium alginate with whey protein concentrate in a 1:2 ratio may provide good prospects for various industrial processing applications [17]. Moreover, gluten, a plant protein consisting of monomeric gliadins and polymeric glutenins, is commonly used for edible packaging films because of its inexpensiveness and abundance and the fact that it is a renewable resource. The use of wheat gluten protein alone to manufacture films resulted in poor mechanical strength, weak water resistance, and susceptibility to rupture [18]. Strawberries wrapped with gluten-edible films had higher visual quality and an acceptable taste for consumers [19], and edible films consisting of gluten combined with polysaccharides [20] and gluten combined with apple pectin [21] had improved physical properties.

Improving the quality of edible films composed of three biopolymers with additional plasticizers, such as glycerol and sorbitol, constitutes another option. Plasticizers are the other component used to generate edible films; they are introduced into film-forming solutions to boost films’ mechanical or structural stability while minimizing permeability. Some common plasticizers include monosaccharides, oligosaccharides, polyols, lipids, and derivatives [22]. Many studies have shown the impacts of plasticizers on the physical, mechanical, and barrier properties of edible films and coatings. Sorbitol and glycerol, often used in edible-film-forming solutions, are largely identical in absorption band areas. However, adding sorbitol and glycerol increases the number of intermolecular hydrogen bonds, and the use of the same plasticizer has varied effects on different film-forming components [1]. Films plasticized with sorbitol are more effective oxygen barriers, homogeneous, and smoother than films plasticized with and without glycerol [23]. Moreover, the properties of polysaccharide-based edible films depend on interactions between polysaccharide chains modified with various polyol plasticizers, as reported in X-ray diffraction, Fourier-transform infrared spectroscopy, and scanning electron microscopy analyses [24]. So far, no research has focused on the production of sodium alginate/gum arabic/gluten edible films to which different types and concentrations of plasticizers have been added. In addition to polymers and plasticizers, the fabrication method is also considered to affect the quality of edible films. The benefits of using the solvent-casting method to create edible films have been documented in numerous studies. This technique involves dissolving polymers, plasticizers, and additives in appropriate solvents and then spreading the solutions into petri dishes or appropriate molds. The edible film is peeled off once it has dried. This method’s benefits include its lower cost and the fact that it can be conducted in a lab without specialized equipment.

In this study, we aimed to produce sodium alginate/gum arabic/gluten edible films and evaluate the effect of glycerol and sorbitol in various concentrations on the physical, mechanical, barrier, and optical properties of these films. To achieve this goal, six edible films corresponding to three levels of glycerol and sorbitol at the same concentrations (2, 4, and 6%) were produced. These samples were characterized in terms of thickness, water solubility, tensile strength, water vapor transmission rate, color, transparency, molecular interactions, and microstructure.

## 2. Materials and Methods

### 2.1. Materials

Gum arabic (Ingredion Sweetener and Starch (Nakhon Ratchasima, Thailand) Co., Ltd), sodium alginate (PT. Sumber Berlian Kimia, Jakarta, Indonesia), and wheat gluten (Gloden Ante, Anhui Ante Food, Co., Ltd., Suzhou, China) were used as the main ingredients in the edible films. Sorbitol and glycerol were used as plasticizers. Span 60 and tween 80, used as emulsifiers, were purchased from a local chemical store (Makassar, Indonesia).

### 2.2. Preparation of Wheat Gluten Solution

Wheat gluten solution was prepared in accordance with an approach used in a previous study, with some modifications [25]. Fifteen grams of wheat gluten was dispersed in 72 mL of ethanol (Merck, Darmstadt, Germany) and then heated using a magnetic stirrer operating at 1200 rpm (DLAB MS-H280-Pro) to a temperature of 50 °C for 10 min. Afterward, 48 mL of distilled water and 0.5 mL of 6 N ammonium hydroxide were added to the wheat gluten solution. The solution was then stirred for 2 min and kept at room temperature.

### 2.3. Preparation of Sodium Alginate and Gum Arabic Solution

Sodium alginate and gum arabic solutions were prepared by mixing 2% (*w*/*v*) sodium alginate and 3% gum arabic (*w*/*v*) in distilled water. The solution was stirred at 80 °C for 30 min. Consequently, a sodium alginate/gum arabic solution was obtained.

### 2.4. Preparation of Edible Film

**Figure 1:**
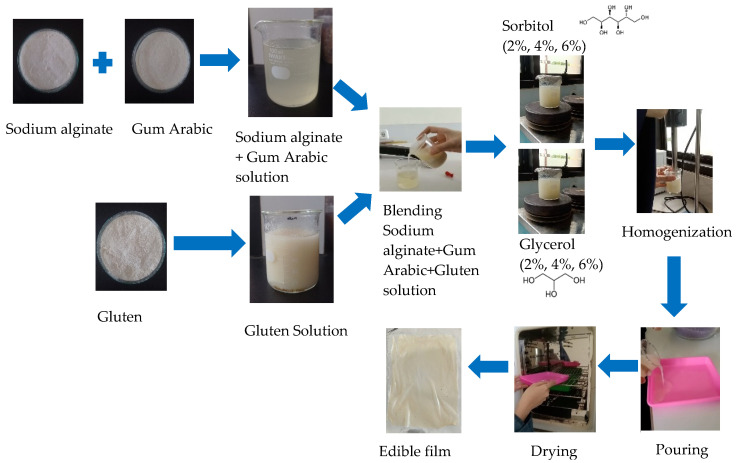
Production of sodium alginate/gum arabic/gluten edible films.

To produce edible films, 10 mL of wheat gluten solution was mixed with 40 mL of sodium alginate and gum arabic solution. Then, tween 80 (0.6 mL) and span 60 (0.4 mL) were dissolved in the solution using a magnetic stirrer. Thereafter, we added sorbitol and glycerol as plasticizers, each at different concentrations (2, 4, and 6% *w*/*v*) at 80 °C and with constant stirring carried out for 30 min. The solution was then homogenized with ultra-turrax (Heidolph RZR 2021, Schwabach, Germany) for 2 min at 24.000 rpm. A total of 100 mL of film-forming solution was poured into a film mold (polypropylene tray, 195 × 145 mm) and oven-dried at 50 °C for 4 h, as shown in Figure 1.

### 2.5. Film Characterization

#### 2.5.1. Thickness

A digital caliper (KRISBOW KW06-422) was used to measure the thickness of individual film samples to the nearest ± 0.01 mm at five randomly selected areas, and the average value was calculated.

#### 2.5.2. Water Solubility

The water solubility of the films was determined according to a method used in a previous study [26], with slight modification. Film samples with dimensions of 2 cm × 2 cm were dried using a drying oven (Huanghua, GP-30BE) at 105 °C for 24 h and weighed to the nearest 0.001 g to determine the initial weight (W1). The dried films were immersed in 50 mL of water for 24 h and then stirred slowly at room temperature. After their immersion, the films were re-dried in an oven at 105 °C for 24 h to determine their final dry weight (W2); at this point, they were insoluble in water. Finally, the water solubility (WS) of the films was calculated using Equation (1):

(1) $$WS\left(\%\right)=\frac{W1-W2}{W1}\;\times 100$$

#### 2.5.3. Tensile Strength

A tensometric testing machine was used to measure the film’s tensile strength. The film was sliced into 20 mm × 40 mm square pieces. The testing equipment was used to clamp the samples and pull them at a speed of 100 mm/min until they broke. Measurements were made at room temperature. Tensile strength is expressed in MPa.

#### 2.5.4. Water Vapor Transmission Rate

The water vapor transmission rate (WVTR) of the film was calculated according to a previous method, with slight modifications [27]. A test cup containing 30 mL of distilled water was employed. Without touching the water, the film was cut into a circle with a 3 cm diameter and placed on the mouth of the test cup. The system’s weight (test cup + water + film) was monitored for 0 to 5 h at 1 h intervals while it was stored in a desiccator containing silica gel. The WVTR was expressed using Equation (2):

(2) $$WVTR=\frac{\Delta W}{\Delta t\cdot A}$$

where ΔW is the amount of water absorbed by the silica gel as a function of time, and A is the area of the film (m^2^), with the slope (ΔW/Δt) of each line being determined by linear regression.

#### 2.5.5. Color

The color of each developed film was determined by measuring its lightness (L*), redness/greenness (a*), and yellowness/blueness (b*) using a colorimeter (CHN Spec CS-10). For each section, measurements were taken three times. The data collected were then averaged.

#### 2.5.6. Transparency

The transparency (T) of the developed films was assessed according to a previous method [28]. Strips of films (1 cm × 3 cm) were supplied. The top and bottom edges of the strips were taped to the surface of a cuvette, and the transmittance at 600 nm was measured with a spectrophotometer (Shimadzu, UV-1280, Kyoto, Japan). Transparency was calculated using Equation (3):

(3) $$T=\frac{A600}{\delta }$$

where T is transparency, A is absorbance, and *δ* is thickness (mm).

#### 2.5.7. Determination of the Films’ Functional Groups

Fourier-transform infrared (FTIR) spectroscopy (QATR-S, Shimadzu, Japan) was used to study potential interactions between the polymers and plasticizers of the films. The range of the spectral region was 4000 cm^−1^ to 500 cm^−1^, with a resolution of 2 cm^−1^ and 32 scans.

#### 2.5.8. Microstructures of the Films

The surfaces of the edible films were examined using a scanning electron microscope (SEM instruments, Tokyo, Japan) at an accelerating voltage of 15 kV. Each sample was placed on a two-sided aluminum plate and covered with gold powder for 30 s. The surfaces of the edible films were examined at 200× magnification.

### 2.6. Data Analysis

In this work, we used statistical analysis to determine the significant differences in thickness, color, solubility, tensile strength, WVTR, and transparency. The mean values and standard deviations of the sodium alginate/gum arabic/gluten films plasticized with glycerol and sorbitol were determined via analysis of variance (ANOVA) using the R software (version 4.1.0, 2021). A *p* value of <0.05 was considered statistically significant. Principal component analysis (PCA), carried out using the *FactoMineR* package (version 2.4), was also performed to evaluate the similarities and differences in the physical, mechanical, barrier, and optical properties between samples.

## 3. Results and Discussion

### 3.1. Thickness of Edible Films

The thickness was measured to assess the edible films’ physical properties, which are related to the volume of film-forming liquid used or the area over which the liquid was dispersed [29]. In this study, the addition of glycerol and sorbitol did not exhibit any significant influences on the thickness, as shown in Table 1, indicating that the thickness properties of the films plasticized with glycerol and sorbitol were not different for each concentration. This result may be due to the inadequate concentrations of the plasticizers, which were insufficient to cause a notable increase in the thicknesses of the films. In line with our results, some authors have reported that increasing the concentration of glycerol from 4 to 6% in wheat gluten films did not lead to a significant difference in thickness [25]. Similarly, it was reported that the use of various glycerol concentrations to create chia seed mucilage films using a solvent-casting method did not significantly affect the thickness properties [30]. Moreover, using various sorbitol concentrations to generate poly(vinyl alcohol) and cationic starch blended films using a casting method did not significantly affect the thickness properties [31]. When the polar groups of sorbitol and glycerol interact with the hydroxyl groups of sodium alginate and glycerol, hydrogen bonds are formed between the polymer chains, forming a polymer chain structure and thus increasing the film’s thickness. This study’s results demonstrate that sodium alginate/gum arabic/gluten plasticized with glycerol and sorbitol could form a thin film within the 0.14–0.17 mm range. Some authors have reported that the ideal thickness of films for food packaging is more than 0.050 mm but less than 0.3 mm [31,32].

### 3.2. Color of the Edible Films

The color of an edible film can influence both the appearance of the product and consumer perceptions. Table 1 shows the color of the sodium alginate/gum arabic/gluten films plasticized with glycerol and sorbitol. The addition of glycerol did not have a significant influence on L*, nor did the addition of sorbitol. The lightness (L*) values of the films plasticized with glycerol ranged from 57.98 to 59.98 and 59.27 to 60.37 for the films plasticized with sorbitol, indicating that the films all had moderate lightness. In this study, none of the lightness values were significantly associated with the thicknesses of the films [33], and the concentrations of both the plasticizers and colorless substances [34] were insufficient to allow light to permeate through the films’ matrices. In the edible film plasticized with glycerol, the a* values ranged from −5.36 to −6.42, whereas values ranging from −4.51 to −7.01 were observed for the edible film plasticized with sorbitol. Plasticizing the edible films with glycerol had no significant influence on the a* values. In contrast, the a* values were found to increase from −6.67 for the films plasticized with 2% sorbitol to −4.51 for the films plasticized with 4% sorbitol; this value decreased to −7.01 for the films plasticized with 6% sorbitol. These results show that the presence of a greenish tint was more common in the films plasticized with sorbitol than those plasticized with glycerol. Moreover, the b* values of the films plasticized with glycerol ranged from 7.66 to 11.11, whereas they ranged from 8.84 to 14.96 for those plasticized with sorbitol. Increasing the sorbitol concentration to 4% and reducing the glycerol concentration to 2% increased the b* values, characteristic of a yellower coloration. Among all the glycerol concentrations, 4% sorbitol appears to have the greatest ability to change the color of a film, an ability that may be related to sorbitol’s high water solubility. Some authors have reported that films based on *Malva sylvestris* flower gum [35] and cellulose-based films [36] loaded with sorbitol tend to be able to provoke changes in b^*^ values to a greater extent than films loaded with glycerol.

### 3.3. Water Solubility of Edible Films

**Figure 2:**
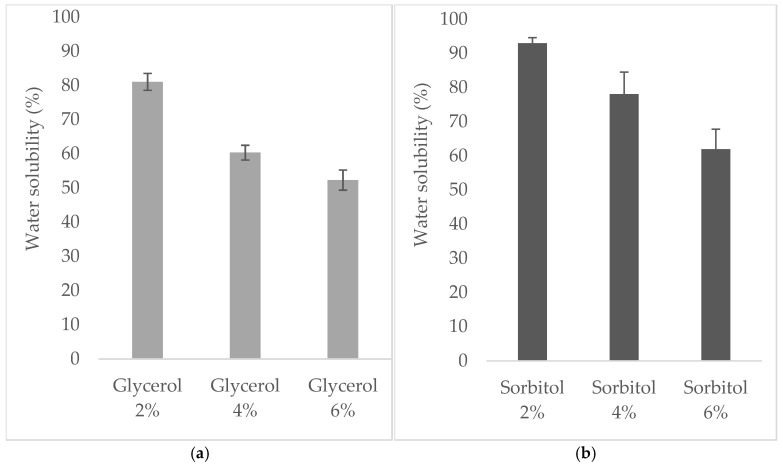
Water solubility of sodium alginate/gum arabic/gluten films plasticized with (**a**) glycerol and (**b**) sorbitol.

A film’s solubility in water is important, since this property is related to the maintenance of product integrity, water resistance for packaging materials, and biodegradability [33,36]. Based on the results shown in Figure 2, the addition of glycerol did not have a significant influence on solubility, nor did the addition of sorbitol. The edible film plasticized with 2% sorbitol had >90% water solubility, but this value decreased with an increase in the sorbitol concentration (Figure 2b), whereas the film plasticized with 2% glycerol had a water solubility of around 80%, but this value also decreased with an increase in the glycerol concentration (Figure 2a). Among the two plasticizers, sorbitol, in each concentration, was found to have the highest solubility in water. Sorbitol has a ring-shaped molecular conformation that may increase the interaction of hydrogen bonds with polymer chains, giving it the highest solubility in water. In the present study, the solubility of the films in water decreased with an increase in the plasticizer concentrations. This phenomenon may have been caused by the presence of gum arabic, which can bind water and form gels [37]. As a result, the mobility of water molecules was restricted, thereby decreasing the overall solubility of substances that would otherwise readily dissolve in water. Additionally, sorbitol and glycerol are water-soluble plasticizers; therefore, adding more of these substances causes the swelling capacity to develop more quickly in the presence of gluten, limiting the flow of water molecules. However, this result contradicts what has been noted in other studies, which reported that plasticizer addition increased water solubility in sweet potato starch [28] and corn-starch-based films [38]. According to these researchers, water solubility increased as the plasticizer concentration increased. Plasticizers can reduce interactions between biopolymer chains and enhance the interaction between the plasticizer and polymer, increasing water solubility. The results of the present study show that the films developed are suitable for food packaging, as they easily dissolve and release the contents. Regarding water solubility, highly water-soluble films might be useful for fresh and minimally processed products [34] and food coating and encapsulation [39].

### 3.4. Tensile Strength of Edible Films

Table 2 shows the tensile strength of the sodium alginate/gum arabic/gluten films plasticized with glycerol and sorbitol. The addition of glycerol and sorbitol had a significant influence on the tensile strength. The edible films plasticized with 2% glycerol and 2% sorbitol had higher tensile strength values than those plasticized with 6% glycerol and 6% sorbitol. The edible films plasticized with glycerol had significantly reduced tensile strengths, falling from 0.10 MPa to 0.01 MPa, and the tensile strength of the edible films plasticized with sorbitol reduced from 0.13 MPa to 0.02 MPa. These findings indicate that the plasticization of sodium alginate/gum arabic/gluten edible films with glycerol led to better tensile strength than plasticization with sorbitol. Increasing the plasticizer concentration will reduce the tensile strength of films by weakening the interactions between the polymer chains. These results are similar to those reported in previous studies, wherein increasing glycerol concentrations reduced the tensile strength of pea-protein-isolate-based films produced by disrupting intramolecular tensions [40], resulting in a decrease in the tensile strength of the films. Meanwhile, we also observed a decrease in tensile strength as the sorbitol concentration increased. These findings align with results reported by a group of researchers who studied the effect of sorbitol on films based on *Dioscorea hispida* starch. These researchers found that sorbitol-plasticized flour films are more resistant to breakage than glycerol-plasticized flour films [41]. These findings indicate that the sodium alginate/gum arabic/gluten edible films plasticized with glycerol had greater tensile strength than those plasticized with sorbitol. This difference may be attributed to the greater molecular weight of sorbitol, making it difficult for the polymer chains to respond to stresses.

### 3.5. Transparency of Edible Films

Transparency is the ability of a material to transmit light. It directly affects consumer acceptability and impacts product appearance. Table 2 indicates the transparency of the sodium alginate/gum arabic/gluten films plasticized with glycerol and sorbitol. Based on these results, the addition of glycerol had a significant influence on the transparency, while the addition of sorbitol did not. The transparency significantly decreased for all the plasticized sodium alginate/gum arabic/gluten (glycerol and sorbitol) films as the plasticizer concentration increased from 2 to 6%. The films plasticized with 2% glycerol and 2% sorbitol had higher transparency values than those plasticized with 4% and 6% glycerol or sorbitol. The data obtained in this study indicate that reduced usage of plasticizers leads to a denser arrangement of polymer chains, lowering the likelihood of light scattering and, thus, enhancing transparency. Compared to the sorbitol-plasticized films, the films plasticized with glycerol showed lower transparency because of their transparent nature and increased glycerol dispersion within the film matrix [42].

### 3.6. Water Vapor Transmission Rate of Edible Films

**Figure 3:**
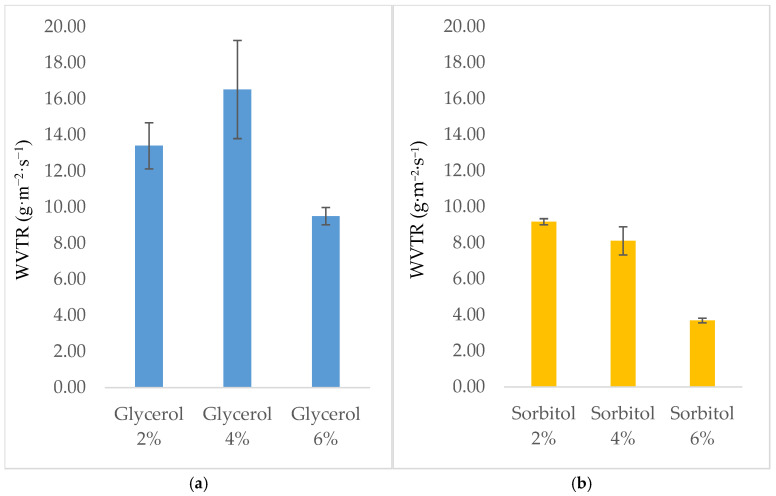
The WVTRs of sodium alginate/gum arabic/gluten films plasticized with (**a**) glycerol and (**b**) sorbitol.

The permeability of water vapor must be minimized, as an edible film or coating is designed to inhibit moisture transfer between food and its surrounding environment or between different components of a heterogeneous food product [25]. Therefore, a high WVTR is the most undesired property of edible film packaging. In this study, the addition of glycerol significantly influenced the WVTR, similar to what was observed when sorbitol was used as a plasticizer, as shown in Figure 3. The WVTR values of the films plasticized with glycerol (Figure 3a) were much higher than those of the films plasticized with sorbitol at the same concentration (Figure 3b), indicating the poor tensile strength properties of the glycerol-containing films, as shown in Table 2. The WVTR values of the films varied between 9.51 g·m^−2^ ·s^−1^ and 16.52 g·m^−2^·s^−1^ when glycerol was added as a plasticizer and between 3.69 g·m^−2^ ·s^−1^ and 9.17 g·m^−2^ ·s^−1^ when the films were plasticized with sorbitol. We also observed that increasing the glycerol or sorbitol concentrations from 2 to 6% decreased the WVTRs and water solubility significantly. This finding could be due to the formation of a network structure between the gum arabic, gluten, and plasticizers that resisted the diffusion of water vapor through the film matrix. The maximum WVTR value was observed when 4% glycerol was used, while the minimum WVTR value was observed when 4% sorbitol was used. This result can be attributed to glycerol’s lower molecular weight (92 Dalton) than sorbitol (192 Dalton). As a result, the addition of glycerol increases the number of active sites by exposing their hydrophilic groups, allowing them to absorb more water [43]. As mentioned by some authors [44], inserting a small hydrophilic molecule of glycerol between adjacent polymeric chains can increase molecular mobility, resulting in a greater interstitial spacing of polymers, thereby facilitating the penetration of water vapor molecules. It is important to note that films must have low WVTRs because they are used for food packaging, especially in considerably humid environments, in order to improve food safety and extending the shelf lives of food products [45].

### 3.7. Fourier-Transform Infrared Analysis of Edible Films

**Figure 4:**
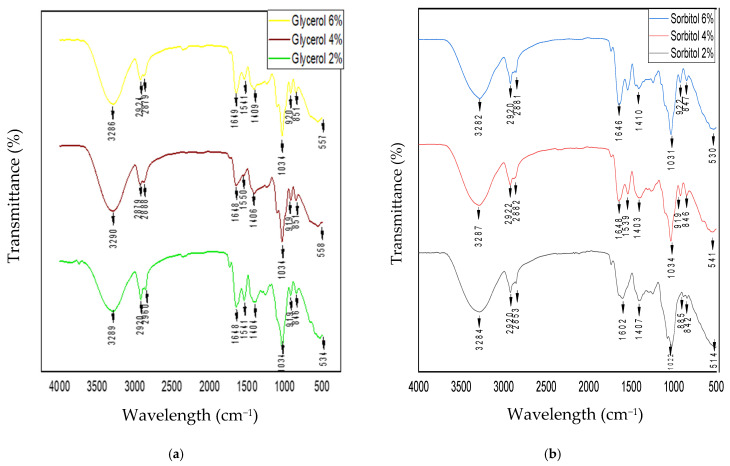
FTIR spectra of sodium alginate/gum arabic/gluten films plasticized with (**a**) glycerol and (**b**) sorbitol.

The FTIR spectra of the films plasticized with glycerol and sorbitol are presented in Figure 4. The spectra of the sodium alginate/gum arabic/gluten films plasticized with glycerol and sorbitol presented almost identical bands due to the vibrational modes of polymer–plasticizer interactions. The films plasticized with 4% glycerol (Figure 4a) and 4% sorbitol (Figure 4b) were observed to exhibit O-H stretching vibrations at 3290 cm^−1^ and 3287 cm^−1^. The absorption peaks of the O-H group for 6% glycerol and 6% sorbitol shifted to lower wavelengths, appearing at 3286 cm^−1^ and 3282 cm^−1^, respectively. This shift suggests the formation of additional hydrogen bonds. The increase in hydrogen bonding may be attributed to the intramolecular and intermolecular interactions between sorbitol and glycerol, both of which possess a greater number of hydroxyl groups [46], in conjunction with polymers. The greater number of absorption bands in the range of 2853–2960 cm^−1^ for the films plasticized with glycerol or sorbitol is related to symmetric and asymmetric C-H vibrations. The results showed that the absorption peaks for all the edible films were in the same region, regardless of the sorbitol and glycerol concentrations, possibly owing to the presence of gluten [47]. Peaks at 1648 cm^−1^ to 1649 cm^−1^ were observed for the films plasticized with glycerol concentrations ranging from 2 to 6%, whereas the peaks for the films plasticized with sorbitol shifted from 1602 cm^−1^ to 1648 cm^−1^. These peaks derived from the asymmetric and symmetrical stretching vibrations of the carboxyl group, indicating good interaction of the plasticizers in the polymer matrix, significantly impacting the mechanical and barrier properties of the films. Furthermore, the peak observed at 1550 cm^−1^ to 1403 cm^−1^ for both films could have formed because of the antisymmetric and symmetric contraction of C-O (carbonyl) in the gum arabic in the film containing gluten. These findings confirm that the presence of gum arabic in the film containing gluten shifted the wavelengths, indicating good incorporation and interaction of the gum arabic in the gluten matrix [20]. Moreover, the broad band at 1034 cm^−1^ to 1022 cm^−1^ was ascribed to the elongation vibration of C-O groups, potentially indicating interactions between the plasticizer used and the polysaccharide structure. The bands of the edible films plasticized with glycerol concentrations ranging from 2% to 6% were consistent, whereas the films plasticized with sorbitol shifted from 1022 cm^−1^ to 1034 cm^−1^, respectively. This finding implies greater interaction between the glycerol and the polysaccharide structure [47]. The results indicate that sodium alginate/gum arabic/gluten is compatible with glycerol and sorbitol, as their spectra were mostly similar in terms of absorption band areas and all the plasticizers were polyols. In line with our study, some researchers have reported that the band areas were similar when glycerol and sorbitol were added to edible-film biopolymers [37,48] 

### 3.8. Morphologies of Edible Films

**Figure 5:**
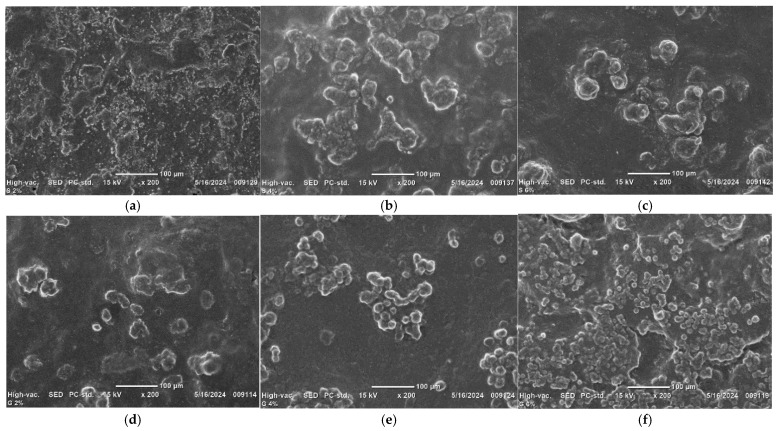
The morphologies of sodium alginate/gum arabic/gluten films plasticized with glycerol and sorbitol: (**a**) 2% sorbitol, (**b**) 4% sorbitol, (**c**) 6% sorbitol, (**d**) 2% glycerol, (**e**) 4% glycerol, and (**f**) 6% glycerol.

The surface morphologies of the edible films plasticized with glycerol and sorbitol in 2–6% concentrations observed using SEM are depicted in Figure 5. The surface morphological structures of the films plasticized with sorbitol and glycerol affected their mechanical properties and WVTRs. The structures of the edible films plasticized with 6% sorbitol (Figure 5c) were less rough and agglomerated than those of the films plasticized with sorbitol at concentrations of 4% (Figure 5b) and 2% (Figure 5a), indicating less there was less dissolution of sorbitol with the increase in the concentration. These findings were confirmed by the results regarding water solubility, which decreased with an increase in the sorbitol concentration. On the contrary, the addition of 6% glycerol to the edible films (Figure 5f) resulted in greater roughness and agglomeration in comparison to the films containing 4% glycerol (Figure 5e) and 2% glycerol (Figure 5d). This finding suggests that a higher concentration of glycerol leads to increased dissolution. We observed that the glycerol was more dissolved than the sorbitol with an increase in the concentration. This finding can be attributed to the natural characteristics of glycerol, which has fewer hydroxyl groups and higher water affinity [49]. These findings are consistent with the tensile strength and WVTR results, likely due to the hydrophilic interaction between the glycerol and polysaccharides, resulting in a lower tensile strength (Table 2) and a higher WVTR (Figure 3a). Moreover, the films to which 6% glycerol was added exhibited greater roughness and agglomeration compared to those modified with 6% sorbitol. This result is associated with a reduction in the tensile strength and an increase in the WVTR, likely due to greater absorption of water. This is because glycerol has a lower molecular weight than sorbitol, facilitating the formation of hydrophilic bonds that enable the absorption of a specific quantity of water [43].

### 3.9. Principal Component Analysis

**Figure 6:**
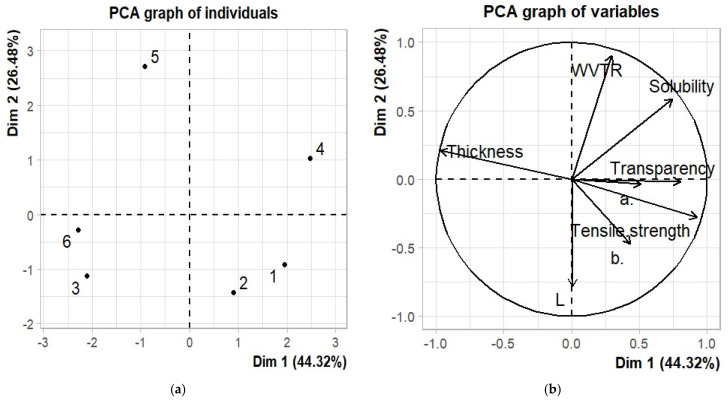
A plot of the principal component analysis of the physical, mechanical, barrier, and optical properties of the sodium alginate/gum arabic/gluten films plasticized with glycerol and sorbitol. The individuals were the six edible films: 1 = sorbitol (2%), 2 = sorbitol (4%), 3 = sorbitol (6%), 4 = glycerol (2%), 5 = glycerol (4%), and 6 = glycerol (6%) (**a**) and the variables were the thickness, color (L, a, and b), transparency, tensile strength, solubility, and WVTR (**b**).

In observing the relationship between the physical, mechanical, barrier, and optical properties of films, a PCA was performed, and a biplot illustrating the information on the samples (Figure 6a) and variables (Figure 6b) was created for the first two dimensions. The PCA results graph explains 70.80% of the variability using the two principal components, with the first component explaining 44.32% and the second component explaining only 26.48% of the variability. The first axis (PC1) describes 44.32% of the variance, mainly assigning positive scores to L, a*, b*, tensile strength, transparency, solubility, and WVTR and negative scores to thickness properties (Figure 6b). The second axis (PC2) describes 26.48% of the variance, with positive scores assigned to main parameters such as thickness, WVTR, and solubility and negative scores assigned to transparency, a*, tensile strength, b*, and L (Figure 6b). On the map of the individual values, the edible films plasticized with 2% glycerol are in the top-right section, suggesting that these films had higher water solubility than the films plasticized with 6% sorbitol. This could be due to the hydrophilic properties of glycerol leading to an increased uptake of water molecules within the films [50]. In contrast, the films plasticized with 4% sorbitol, located in the bottom-right section of the map, exhibited a lower water vapor transmission rate (WVTR) than those plasticized with 4% glycerol. This difference can be explained by the characteristics of sorbitol, which has a greater molecular weight (192 Dalton) and twice the number of hydroxyl groups than glycerol. These attributes indicate that sorbitol forms more intramolecular hydrogen bonds between neighboring polymer chains. As a result, the space between inter-cellulose chains within the film network is diminished, leading to a reduced rate of water diffusion and, consequently, a lower WVTR. A similar trend was observed in cellulose-based films plasticized with sorbitol and glycerol [36].

## 4. Conclusions

A sodium alginate/gum arabic/gluten film-forming solution is an interesting ingredient for the fabrication of edible films. This work shows the feasibility of preparing sodium alginate/gum arabic/gluten film-forming solutions plasticized with sorbitol and glycerol. The impacts of three different concentrations of sorbitol and glycerol showed that they were ideal for creating films with adequate thickness and moderate lightness. However, the greenness and yellowness changed significantly with the increase in the sorbitol concentration. Although the tensile strength of the edible films plasticized with sorbitol was higher than that of the glycerol-plasticized films, the latter films were more transparent. The water solubility and WVTRs of the films decreased significantly upon increasing the sorbitol and glycerol concentrations. Additionally, sorbitol and glycerol addition affected the surface morphologies of the films, and FTIR analysis showed that both plasticizers had similar patterns of absorption band areas, indicating that sodium alginate/gum arabic/gluten engages in good interactions with sorbitol and glycerol. Using the films from this study could be a viable alternative for packaging fresh and minimally processed products. Accordingly, we recommend further exploration of the development of active packaging for edible packaging based on sodium alginate, gum arabic, and gluten.

## Figures and Tables

**Table 1:** Thickness and color of sodium alginate/gum arabic/gluten films plasticized with glycerol and sorbitol.

Samples	Thickness (mm)	Color		
		L*	a*	b*
Glycerol				
Glycerol 2%	0.14 ± 0.01 ^a^	58.66 ± 0.64 ^a^	−5.36 ± 0.20 ^a^	11.11 ± 1.26 ^a^
Glycerol 4%	0.17 ± 0.02 ^a^	57.98 ± 0.41 ^a^	−6.24 ± 0.72 ^a^	7.66 ± 3.41 ^b^
Glycerol 6%	0.17 ± 0.02 ^a^	59.98 ± 4.30 ^a^	−6.42 ± 0.38 ^a^	8.62 ± 1.30 ^a^
Sorbitol				
Sorbitol 2%	0.14 ± 0.01 ^a^	60.37 ± 0.72 ^a^	−6.67 ± 0.15 ^a^	8.84 ± 0.29 ^a^
Sorbitol 4%	0.15 ± 0.01 ^a^	59.27 ± 0.69 ^a^	−4.51 ± 0.42 ^b^	14.96 ± 1.52 ^b^
Sorbitol 6%	0.17 ± 0.01 ^a^	59.33 ± 0.42 ^a^	−7.01 ± 0.27 ^a^	9.19 ± 0.43 ^a^

Data are the means ± standard deviations. Values in the same column with the same superscript letter were not statistically significant with respect to each other (*p* > 0.05).

**Table 2:** The tensile strength and transparency of sodium alginate/gum arabic/gluten films plasticized with glycerol and sorbitol.

Samples	Tensile Strength (MPa)	Transparency
Glycerol		
Glycerol 2%	0.10 ± 0.00 ^a^	17.85 ± 1.07 ^a^
Glycerol 4%	0.01 ± 0.01 ^b^	13.58 ± 0.73 ^b^
Glycerol 6%	0.01 ± 0.00 ^b^	6.39 ± 0.35 ^c^
Sorbitol		
Sorbitol 2%	0.13 ± 0.07 ^a^	20.89 ± 2.57 ^a^
Sorbitol 4%	0.07 ± 0.03 ^a^	13.61 ± 0.81 ^b^
Sorbitol 6%	0.02 ± 0.01 ^b^	13.91 ± 0.83 ^b^

Data are the means ± standard deviations. Values in the same column with the same superscript letter were not statistically significantly different from each other (*p* > 0.05).

## Data Availability

The original contributions presented in this study are included in the article; further inquiries can be directed to the corresponding author.
